# Vegetable‐enriched bread: Pilot and feasibility study of measurement of changes in skin carotenoid concentrations by reflection spectroscopy as a biomarker of vegetable intake

**DOI:** 10.1002/fsn3.3327

**Published:** 2023-03-22

**Authors:** Isaac Amoah, Carolyn Cairncross, Elaine Rush

**Affiliations:** ^1^ Faculty of Health and Environmental Sciences Auckland University of Technology Auckland 1010 New Zealand; ^2^ Riddet Institute, Massey University Private Bag 11222 Palmerston North 4442 New Zealand; ^3^ Department of Biochemistry and Biotechnology Kwame Nkrumah University of Science and Technology Kumasi Ghana

**Keywords:** bread, carotenoid reflection scores, fruits, plasma carotenoid concentration, vegetables

## Abstract

Globally, bread is a staple food and thus a promising vehicle for the delivery of nutrients from vegetables including carotenoids. The aim of this pilot/feasibility, pre–post experimental study was to measure skin (Veggie Meter™) and plasma carotenoid concentrations 1 week before (week −1), immediately prior to (week 0), and after (week 2) 14 days of daily consumption of 200 g pumpkin‐ and sweetcorn‐enriched bread (VB). At each measurement point, total vegetable and fruit intake and specific carotenoid‐rich foods were assessed by questionnaire. Participants (*n* = 10, 8 males, 2 females) were aged between 19 and 39 years and weighed 90 ± 20 kg. Vegetable and fruit intake was low and less than one serving/day of foods containing carotenoids. Prior to the intervention, measures of carotenoid‐containing foods and skin or plasma carotenoids were not different when measured a week apart. Consumption of the VB did not result in statistically significant changes in either the skin or plasma carotenoid measurements. Plasma carotenoid concentrations and the carotenoid reflection scores had a large and positive (*r* = .845, 95% CI 0.697, 0.924) association. The relationship between the number of servings of carotenoid‐rich foods with the plasma carotenoid and carotenoid reflection scores was positive and of moderate strength. In conclusion, carotenoid status was not measurably changed with the consumption of 200 g VB each day for 2 weeks. Subjective carotenoid‐rich food intake was positively associated with objective biomarkers of carotenoids. The Veggie meter™ has the potential to provide portable measurement of circulating carotenoids and be indicative of intake of carotenoid‐rich foods.

## Graphical abstract

Plasma carotenoid concentrations and the carotenoid reflection scores had a large and positive association. Carotenoid status was not measurably changed with the consumption of 200 g vegetable‐enriched bread each day for 2 weeks. The Veggie meter™ has the potential to provide portable measurement of circulating carotenoids and be indicative of intake of carotenoid‐rich foods.

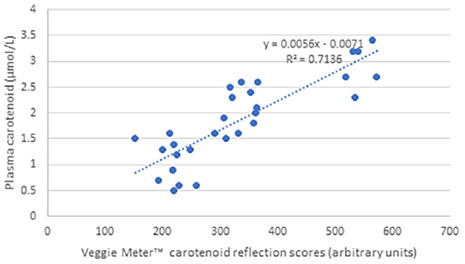

## 1 INTRODUCTION

Bread is consumed as a common staple food worldwide (Kourkouta et al., 2017). Enrichment of bread with vegetables can increase the nutrient density of certain important nutrients including carotenoids (Ranawana et al., 2016). The addition of vegetables and seeds to frequently consumed foods as part of recipe development improves the quantity of bioactive nutrients, particularly the carotenoid concentration, increases variety, and has the potential to demonstrate health‐promoting benefits (Amoah et al., 2022).

Carotenoids as antioxidants are essential to the human body due to their role in complementing the body's inherent antioxidant stores through inactivating free radicals, and some are precursors of vitamin A (Elvira‐Torales et al., 2019; Moran et al., 2018). The main dietary sources of carotenoids are dark green and orange vegetables and fruit and they are also present in animal products including dairy, eggs, and liver (Ashton et al., 2017; Pezdirc et al., 2016). Plasma carotenoid concentration is a biomarker of vegetable and fruit intake (Campbell et al., 1994) which determines the carotenoid status of the body. Carotenoids that are fat soluble in nature are shuttled by blood to storage sites including tissues of the skin (Ashton et al., 2017). The “gold standard” for carotenoid status evaluation involves the assessment of plasma or serum carotenoid concentration. This, however, comes with its attendant challenges including limiting participants' participation in research studies because a venepuncture is required, cost‐intensive nature, and is time‐consuming. A portable “Veggie meter”™ (Longevity Link Corporation, Utah) that non‐invasively employs the principle of reflection spectroscopy of the fat pad of the index finger evaluates the concentration of skin carotenoid as a score and has been reported in a study involving 54 participants to be a reliable (SD 3.4%, 4.1%) and valid (*r* = .81) device for the assessment of concentration of carotenoids (Ermakov et al., 2018). Validation and measurement of reliability of the Veggie meter™ in different populations allow researchers conducting larger studies of dietary interventions to objectively investigate the impact of consumption of foods rich in carotenoids such as vegetables and fruits on the storage profile of carotenoid stores in the body.

It has previously been shown (Amoah et al., 2020) that enrichment of bread with flaxseed and drum‐dried flours from pumpkin and sweetcorn compared to white and wheatmeal bread resulted in bread with appealing organoleptic attributes and favorable textural properties that were easier to swallow in older adults (Amoah et al., 2020). In addition, compared to white and wheatmeal breads, the vegetable‐enriched bread attenuated insulin response and imparted enhanced sensation of fullness for 2 h after consumption (Amoah et al., 2021). Pumpkins and sweetcorn are good dietary sources of the carotenoids β‐carotene and lutein (Elvira‐Torales et al., 2019). What is unknown, however, is the quantity (how much) and duration (for how long) it would take for the vegetable‐enriched bread to impact changes in biomarkers of carotenoid when the bread is consumed.

The aim of this pilot/feasibility, pre–post experimental study was to measure skin (Veggie Meter™) and plasma carotenoid concentrations 1 week before (week −1), immediately prior to (week 0), and after (week 2) 14 days of daily consumption of 200 g pumpkin‐ and sweetcorn‐enriched bread. The association of carotenoid status with vegetable and fruit consumption by two standard questions and a 6‐item carotenoid food frequency questionnaire was also investigated.

## 2 METHOD

### 2.1 Study design

This feasibility and pilot study first assessed if participants would comply with measurements and eat the bread every day, and second was to inform the design of a larger study including the dose of vegetable‐enriched bread that would be required for meaningful change in carotenoid status. A sample size of 10 was pragmatically determined to be adequate to meet these objectives (Bell et al., 2018).

The study took place in December 2018 at the Nutrition and Metabolism Laboratory which is located at the South Campus of the Auckland University of Technology. Ethical approval was obtained from the Auckland University of Technology Ethics Committee (AUTEC, approval number 18/146). The trial was registered with the Australian New Zealand Clinical Trial Registry (reference ID: ACTRN12618002040246).

The researcher provided explanations (verbal and written) of the study to the participants who then signed informed consent. The study had these stipulated inclusion criteria: participants who were regular bread consumers (at least three times a week), were willing to consume four slices (200 g) of the vegetable‐enriched bread every day for 2 weeks, and were 18 years or older in age. Participants who had liver disease, were allergic to gluten or any of the ingredients in the bread or who were consuming supplements containing carotenoids were excluded from the study.

The participants visited the laboratory 3 separate times, involving two baseline measures (weeks −1 and 0) with a week apart, and made a final visit after the intervention (week 2). The weight of the participants (wearing light clothing) was determined to the nearest 0.1 kg using a calibrated Soehnle bathroom scale. At each visit, participant's blood was sampled, skin carotenoid score was measured with the Veggie meter™, and a short food frequency questionnaire was completed.

### 2.2 Dietary assessment

The food frequency questionnaire had two standard vegetable and fruit frequency questions stipulated in the New Zealand National Health Survey (Ministry of Health, 2011). The other part of the questionnaire contained specific carotenoid‐rich foods as indicated in the New Zealand Food Composition Tables (Plant & Food Research & Ministry of Health, 2016). The specific carotenoid‐rich foods included egg, pumpkin, carrots, liver, green leafy vegetables including spinach and kale, yogurt, milk, and cheese. Participants completed the food frequency questionnaire at weeks −1, 0, and 2.

Standardized serving sizes were defined on the questionnaire. Servings of carotenoid foods consumed in a week were calculated by summing the weekly servings of all the carotenoid‐rich foods.

During the participants' second laboratory visit (week 0), 14× 200 g (four standard slices per day) portions of bread enriched with vegetables were given to them for consumption over the 2‐week trial period. The bread proportions were prepared from a batch baked prior to the intervention. The bread was handed to the participants frozen at −20°C and wrapped in aluminum foil. A preliminary trial conducted by the researcher established that defrosting the vegetable‐enriched bread for 1 h was enough to maintain the organoleptic integrity of the bread. This information was conveyed to the participants and they were asked to defrost the frozen bread for 1 h prior to consumption. In the case of participants with limited storage space in their refrigerator, they took 1 week supply and collected the remainder on day 7 of the intervention period. In order to keep track of the participants' compliance in regards to the vegetable‐enriched bread intake, the participants were provided with an intervention compliance diary to record date and time of consumption of the 200 g portions of the bread over the 2‐week intervention period. A routine follow‐up on participants in the form of phone calls to encourage compliance and provide support was carried out by the researcher at least two times during the trial period.

### 2.3 Bread preparation and analysis

The vegetable‐enriched bread was produced in the Auckland University of Technology School of Hospitality kitchens. The procedure and recipe for bread preparation have been extensively indicated in our earlier published work (Amoah et al., 2021). Briefly, the ingredients used in the development of vegetable‐enriched bread were strong white wheat flour, wholemeal wheat flour, whole flaxseed, sprouted wheat flour, pumpkin flour, sweetcorn flour, fresh yeast, and salt. The indirect method was employed for the bread‐making process. The vegetable‐enriched bread was analyzed by AsureQuality, an ISO‐registered laboratory, and was reported to contain 237 μg/100 g β‐carotene (Amoah et al., 2021).

### 2.4 Dosage of vegetable‐enriched bread

The 200 g (four standard slices) of the vegetable‐enriched bread consumed daily was calculated to contain 474 μg β‐carotene (79 μg retinol equivalents). This is equivalent to 16% of the recommended dietary intake (RDI) for women (14 + years and above) (700 μg) and 10% of the RDI for men (14 + years and above) (900 μg) (National Health and Medical Research Council (Australia), 2006).

### 2.5 Biochemical assessments

A 5 mL sample of venous blood from the participants obtained in non‐fasted state was collected into a 10 mL BD Vacutainer® EDTA tube (Becton Dickinson) for plasma carotenoid concentration measurement at weeks −1, 0, and 2. Prevention of carotenoid degradation by light was ensured by wrapping the glass tubes with aluminum foil. After centrifugation at 1500 revs/min at 4°C for 10 min, plasma was removed and stored in Eppendorf tubes at −80°C. For analysis of the plasma carotenoid concentration, the frozen samples were couriered at −80°C to IANZ‐accredited Canterbury Health Laboratories (CHL). Analysis of the plasma carotenoid proceeded with the precipitation of plasma proteins through the addition of ethyl alcohol and extraction of the carotenoids into petroleum ether. A spectrophotometer (Cary 4000, Agilent) was used to measure carotenoids colorimetrically using a wavelength of 440 nm. At this wavelength, β‐carotene, lycopene, lutein, zeaxanthin, β‐crytoxanthin, and all isomers of these compounds are detected. The method was reported as linear up to 20 μmol/L and the lowest measurable carotenoid concentration using this method was 0.1 μmol/L. The laboratory stipulates “normal” carotenoid concentration of 1.5–3.0 μmol/L as the acceptable reference range, with the current coefficient of variation of the measurement as 4.4% (Sies, 2019).

### 2.6 Skin carotenoid assessment

Following the manufacturer's instructions, the point‐of‐care Veggie meter™ (Longevity Link Corporation) was calibrated with white and black references. The participants washed their hands with warm water and soap, rinsed them well, and used a paper towel to dry them. To evaluate the participants' skin carotenoid score, the tip of the right index finger was placed on the Veggie meter™ device's light lens, light pressure was applied, and the fat pad was scanned at least three times. The average of the triplicate skin carotenoid scores was recorded for each participant. The range of possible scores on the Veggie meter™ is 0–800 with an acceptable precision between the three scans with an allowable variation among scans of <10%.

### 2.7 Statistical analysis

The normality of the data was checked using the Shapiro–Wilk test (where *p* > .05 implied normality for the data). Line charts and scatter plots were employed to visualize the data. Except for intake of egg, intake of liver, baseline daily vegetable intake (week 0), carrot/pumpkin intake (week 2), skin carotenoid score (week −1), green leafy vegetable intake, and average baseline Veggie meter™ carotenoid score, all the data generally followed a normal distribution. Frequency of daily intake of fruit and vegetables, carrot/pumpkin, eggs, green leafy vegetables, and dairy products were reported using descriptive statistics. The residuals in one‐way ANOVA were used to determine intra‐individual coefficient of variation (CV) between weeks −1 and 0. Individual baseline measures (weeks −1 and 0) were compared (paired *t*‐test), and if not different, averaged. Correlations between skin carotenoid reflection scores, plasma carotenoid concentration, and frequency of intake of carotenoid‐rich foods were explored using Pearson r. Spearman correlation was used in instances where associations were established between a normally distributed variable and a non‐normally distributed variable. The magnitudes of the correlation coefficients with 95% CV were interpreted using Cohen's scale (Cohen, 1988): <0.10, trivial; 0.10–0.29, small; 0.30–0.49, moderate; and ≥0.50, large. Kendall's tau‐b correlation was used if variables were both non‐normally distributed. Interpretation of the magnitude of effect size of the change in the post‐treatment measure from the baseline measures was carried out using Cohen's effect size system with <0.2 categorized as trivial, 0.2 small, 0.5 moderate, and 0.8 large (Field, 2009). Wilcoxon sign test was used to test for differences in the weekly median consumption of carotenoid‐rich foods. A regression equation was established between the plasma carotenoid concentration and the Veggie Meter™ carotenoid reflection scores. Unless otherwise stated, all data were analyzed using International Business Machines Corporation® SPSS® Statistics Version 25.

## 3 RESULTS

### 3.1 Baseline

Male (*n* = 10, male = 8, female = 2) was the predominant gender among the participants, with weight ranging between 61 and 125 kg (mean ± SD; 90 ± 20 kg), and they were relatively young (mean age 30 years, median, 25th, and 75th: 30, 19, and 39). The participants were students attending a university in a high‐deprivation location in Auckland, New Zealand.

At weeks −1 and 0, the median number of servings of vegetables plus fruit each day was 4 with an intra‐quartile range (IQR) for vegetables from 2.8 to 5.3 and for fruit from 1.75 to 5.0 (Table 1). Green leafy vegetable consumption was low, with a median of 1 serving for weeks −1 and 0. At weeks −1 and 0, the median weekly intake of total carotenoid from carotenoid‐rich foods was 5.7 (IQR 3.8, 6.7) and 6.4 (IQR 3.0, 6.8) servings, respectively. The average median weekly intake of carotenoid foods at baseline was 6.1 (IQR 3.6, 6.6) servings (Table 1), which is less than one serving a day. Overall, the baseline measures (weeks −1 and 0) of carotenoid concentrations and fruit and vegetables and carotenoid food intake were not different, and therefore the two baseline measures were averaged to compare with the post‐intervention measure. Intra‐individual variation of measures a week apart was low.

**TABLE 1:** Baseline frequencies of serving consumption of foods containing carotenoids.

	Frequency of consumption		
Food	Week −1	Week 0	Average of week −1 and 0
Fruit/day	1.5 (1.0, 2.3)	2.0 (0.75, 2.3)	1.5 (1.0, 2.3) [0, 4]
Vegetables/day	2.0 (1.8, 2.3)	2.0 (1.0, 3.0)	2.0 (1.4, 2.6) [0.5, 3.5]
Fruit and vegetables/day	4.0 (2.8, 5.3)	4.0 (1.75, 5.0)	4.0 (2.4, 5.1) [0.5, 6.0]
Carrot or pumpkin/week	1.5 (0.4, 3.3)	0.63 (0.0, 3.0)^a^	1.0 (0.3, 3.1) [0, 4]
Eggs/week	3.0 (2.5, 4.9)	3.0 (2.5, 4.9)	3.0 (2.5, 4.9) [0.25, 14.0]
Green leafy vegetables/week	0.5 (0.0, 3.5)	1.5 (0.0, 4.3)	1.0 (0.0, 3.5) [0, 5]
Dairy/week	3.0 (2.0, 5.0)	3.3 (1.0, 4.3)	3.0 (2.0, 5.0) [2, 6.0]
Total carotenoid foods/week^a^	5.7 (3.8, 6.7)	6.4 (3.0, 6.8)	6.1 (3.6, 6.6) [1.1, 8.3]

*Note*: Data are given as median (25th and 75th percentile) [Range].

^a^ Significantly different by Wilcoxon sign test.

Baseline mean plasma carotenoid concentration at week −1 was 1.8 ± 0.9 μmol/L, and 1.8 ± 0.8 μmol/L at week 0, with the mean difference 0.02 μmol/L (95% CI −0.2, 0.2) and intra‐individual coefficient of variation 9%. The mean Veggie meter™ carotenoid reflection score was 334 ± 130 at week −1 and 335 ± 126 at week 0, and the mean difference − 1 (95% CI −18, 17) with an intra‐individual coefficient of variation of 5%. Pearson r correlation between the baseline plasma carotenoid concentrations was 0.961, and the baseline means carotenoid reflection score was 0.981.

### 3.2 Intervention

The majority of the study participants reported consumption of the bread in the evening, with times ranging from 6:00 p.m. to 11:00 p.m. Compliance was 100%, as each of the participants reported consuming 200 g of the vegetable‐enriched bread each day over the 2‐week period and all participants reported on time and completed all measurements.

### 3.3 Validation of veggie meter™ skin carotenoid reflection scores

**FIGURE 1:**
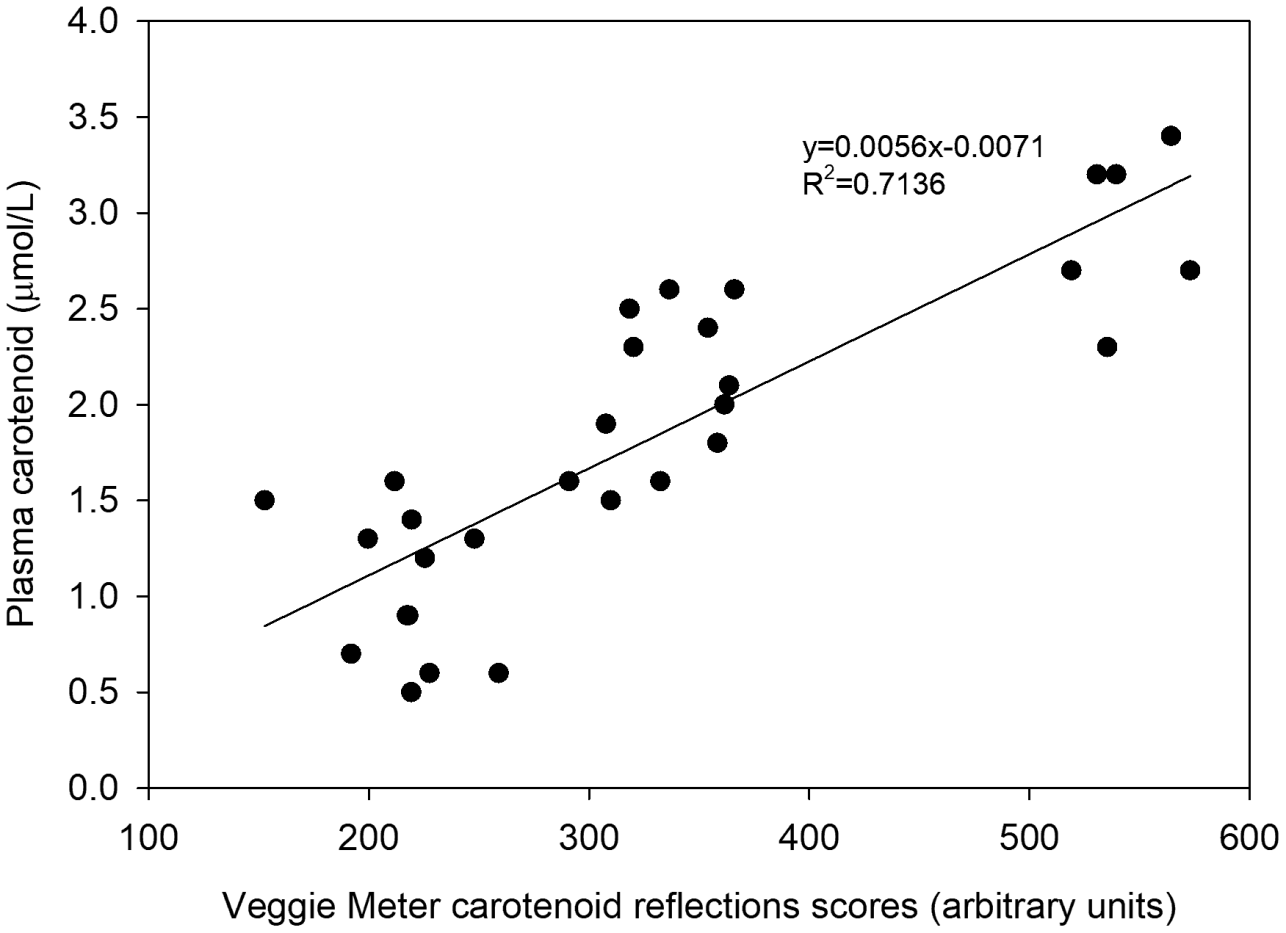
Association between non‐invasive skin carotenoid scores and invasive plasma carotenoid measure.

Thirty measures of plasma carotenoid concentration measures and Veggie meter™ carotenoid reflection score were available from the three measurement points (weeks −1, 0, and 2). A strong agreement was found between the plasma carotenoid concentrations and the skin carotenoid reflection scores (Table 2 and Figure 1). Separately all three time point measures of association demonstrated that a large proportion (72% (*r* ^2^ overall 95% CI 49, 85%)) of the variation in the relationship between the two methods was explained. The regression equation to determine plasma carotenoid concentration from carotenoid reflection score was as follows: plasma carotenoid concentration (μmol/L) = 0.0056 × carotenoid reflection score = −0.0071, *r* ^2^ .72 (Figure 1).

**TABLE 2:** Associations between Veggie meter™ carotenoid reflection scores and plasma carotenoid measures.

Time points	Pearson *r* (95% CI)			
	Week −1 (*n* = 10)	Week 0 (*n* = 10)	Week 2 (*n* = 10)	Combined (*n* = 30)
Week −1 (*n* = 10)	0.842^a^ (0.452, 0.962)			
Week 0 (*n* = 10)		0.864^a^ (0.514, 0.967)		
Week 2 (*n* = 10)			0.842^a^ (0.452, 0.962)	
Combined (*n* = 30)				0.845^a^ (0.697, 0.924)

*Note*: Combined data from all the participants' visits (two baselines and one post‐intervention).

Abbreviation: CI, confidence interval.

^a^ Significant association between the Veggie meter™ skin carotenoid reflection scores and the plasma carotenoid measures.

The total weekly servings of carotenoid foods were positively correlated with both the plasma carotenoid concentration and carotenoid reflection scores. The strength of the correlation for the carotenoid reflection scores (0.690 and 0.687) was generally of a higher magnitude than the plasma carotenoid concentrations (0.533 and 0.455) and was statistically significant at weeks −1 and 0 (Table 3). Negative and statistically significant correlation of participants' weight with plasma carotenoid concentration at weeks −1 and 2, and week 0 with the carotenoid reflection score were also observed (Table 3).

**TABLE 3:** Associations between total servings of carotenoid‐rich foods and plasma carotenoid concentration and carotenoid reflection score.

	Correlation coefficient (95% CI)					
	Plasma carotenoid week −1	Plasma carotenoid week 0	Plasma carotenoid week 2	VM carotenoid reflection score week −1	VM carotenoid reflection score week 0	VM carotenoid reflection score week 2
Total servings of carotenoid foods/week	0.533 (−0.145, 0.870)	0.455 (−0.245, 0.843)	0.513 (−0.172, 0.864)	**0**.**690** (**0**.**107**, **0**.**920**)	**0**.**687** (**0**.**101**, **0**.**919**)	0.612 (−0.029, 0.896)
Body weight	−**0**.**647** (**−0**.**907**, **−0**.**029**)	−0.620 (−0.893, 0.045)	−**0.681** (**−0.917**, **−0.090**)	−0.512 (−0.863, 0.174)	−**0.754** ^a^ (**−0.938**, **−0.237**)	−0.565 (−0.881, 0.100)

*Note*: Bold text indicates correlation is significant at 95% CI.

Abbreviation: CI, confidence interval.

^a^ Spearman correlation. All other results presented are outcome of the Pearson r correlation.

When the participants' daily fruit and vegetable serves from weeks −1 and 0 were averaged, the strength of association increased, particularly with the carotenoid skin reflection measures. The magnitude of the positive correlation coefficients for the plasma carotenoid concentrations and skin carotenoid reflection scores were moderate to large (Table 4).

**TABLE 4:** Association of carotenoid‐rich food intake (fruits and vegetables) with biomarker measures of carotenoid status (*n* = 10).

	Fruit week −1	Fruit week 0	Fruit Av	Fruit week 2	Veg week −1	Veg week 0	Veg Av	Veg week 2	Fruit & veg week −1	Fruit & veg week 0	Fruit & veg Av	Fruit & veg week 2
Plasma carotenoid measure												
Week −1	0.320				0.308				0.396			
Week 0		0.304				0.508				0.462		
Baseline Av			0.326				0.446				0.457	
Week 2				0.514				0.255				0.419
Skin carotenoid reflection scores												
Week −1	0.506				0.402				0.580			
Week 0		0.319^a^				**0.547** ^a^				0.632		
Baseline Av			0.377^a^				0.489^a^				**0.598** ^a^	
Week 2			0.463^b^	**0.676**			**0.689** ^b^	0.459			**0.755** ^b^	0.624

*Note*: Bold text indicates correlation is statistically significant at 95% CI. Fruit, vegetable, and fruit and vegetable consumption were reported in daily intakes.

Abbreviations: Av, average; Veg, vegetables.

^a^ Kendall correlation.

^b^ Spearman correlation. All other results presented are outcome of the Pearson r correlation.

Weekly (week 2) consumption of animal‐based carotenoid‐containing foods (milk, cheese, yogurt, and liver) had a positive correlation with the plasma carotenoid measures and skin carotenoid reflection scores. The correlations were, however, small to moderate (*r* = .322, 95%CI −0.386, 0.791) and (*r* = .401, 95% CI −0.306, 0.823), respectively. A similar observation was recorded for liver (week 2) with correlations of (*r* = .295, 95% CI −0.411, 0.780) and (*r* = .245, 95% CI −0.455, 0.758), respectively.

There was a trivial difference in plasma carotenoid concentration after the intervention (*p* = .370) compared with baseline. A similar observation was made for the skin carotenoid scores (*p* = .117) (Table 5).

**TABLE 5:** Changes between averaged baseline and post‐intervention carotenoid measurement for 10 participants.

Measure	Mean (*n* = 10)	Standard deviation	Change	95% CI	*p*‐value
Baseline plasma carotenoid, μmol/L	1.81	0.84	−0.06	(−0.204, 0.084)	.370
Post‐treatment plasma carotenoid, μmol/L	1.87	0.85			
Baseline skin carotenoid score	334.10	127.65	16.37	(−4.984, 37.724)	.117
Post‐treatment skin carotenoid score	318.20	129.01			

## 4 DISCUSSION

Compliance with and acceptability of the intervention and the measurements were feasible in the setting of this small study with students at a university in a high‐deprivation location in Auckland, New Zealand. The 10 participants consumed substantially less servings of vegetables and fruits than the dietary guidelines of five vegetables and two fruits a day (Ministry of Health, 2022). A serving of any carotenoid‐containing foods was consumed less than once a week, suggesting low dietary diversity in the participants' dietary patterns. Plasma and skin biomarkers of carotenoid status on average were also low, which is in line with the low intake. Evidence is provided for the validity of the portable Veggie meter™ reflection spectroscopy scores as a pragmatic field measurement of vegetable and carotenoid‐containing food intake. Ermakov et al. (2018) validated carotenoid scores from the Veggie meter™ with serum carotenoids of participants and observed a strong association (*r* = .81; *p* < .001) with 54 participants. Similarly, we found a strong association (*r* ^2^ = .72) of the laboratory measure with reflection spectroscopy in this present study. This study confirms that the Veggie meter™ is a convenient device that measures an individual's carotenoid score non‐invasively and has the potential to be used in large population‐based studies to track vegetable and fruit intake of the population during interventions without the need to sample blood or pay for analysis of plasma carotenoids.

The 2‐week intake of vegetable‐enriched bread (200 g/day) resulted in no measurable plasma carotenoid change. Factors including that the dose of carotenoid in the vegetable‐enriched bread consumed over the 2‐week treatment period was insufficient to change participant's storage status may have contributed to this null finding. In addition, the negative association between participants' body weight and their carotenoid stores was measured by plasma carotenoid concentration. This implies that as the weight of the participants increased, the carotenoid dose required to cause change in concentration would expectantly also increase (Rush et al., 2020), probably due to an increase in fat mass. Participants' body weight in this study was above the average considered for recommendations for dietary intake. In a more practical perspective, participants with lower body weight will relatively require lower amount of vegetable‐enriched bread to cause body carotenoid change compared to participants with higher body weight and thus the dose of carotenoid should be calibrated to body size. Conversely, participants in the present study consumed equal amounts of vegetable‐enriched bread, which may have possibly contributed to the lack of change in the carotenoid stores. Factors, including dose size (higher carotenoid dosages) (Müller et al., 1999) and the duration of consumption of carotenoid‐rich foods, have been reported as covariates in previous studies. It has been reported that serum concentration reflects the increase in carotenoid intake by 2 weeks (Jahns et al., 2014) to 12 weeks (Ermakov & Gellermann, 2012), but skin carotenoid concentrations may take longer to show the effect of intervention (Obana et al., 2020). Specifically, Obana et al. (2020) found that a significant increase in skin carotenoid concentration started at 4 weeks and continued to increase to 16 weeks with the daily consumption of a supplement containing 20 mg lutein and 4 mg zeaxanthin. The duration of the present 2 week study was short and if longer may have shown an effect. Vegetable‐enriched bread plus dietary counseling to increase vegetable intake could also increase the effect of the intervention and be more sustainable.

The use of bakery products including bread as a carrier medium for carotenoid and vegetable delivery is challenging. An increase in the concentration of carotenoid in bread would require the addition of higher amounts of ingredients rich in carotenoids including pumpkin, carrot, and tomato powders. These ingredients would impact the physical qualities of the bread including its loaf volume and texture; and its organoleptic attributes, including color, which negatively may be perceived by consumers. For example, in an earlier study (Nour et al., 2015) where bread was enriched with tomato products (rich source of lycopene) as a way of increasing its carotenoid profile, the authors reported lower liking of the bread, especially as the tomato enrichment increased. Additionally, the physical qualities of the bread including its specific loaf volume were adversely impacted (Nour et al., 2015). Even though pumpkin and sweetcorn used in our novel bread are good sources of carotenoids including β‐carotenes and lutein (Elvira‐Torales et al., 2019), these essential nutrients are heat labile and consequently may be degraded during baking (Kopec & Failla, 2018). The degree to which degradation may have occurred is unknown in this study. The application of encapsulation technology of bioactives including carotenoid using, for example, maltodextrin as the cell wall material has been proposed as a prominent strategy to adopt toward enhancing the stability of these heat‐labile compounds during bread baking (Amoah et al., 2018). Interestingly, due to the carbohydrate nature of most encapsulating wall materials such as maltodextrin, which is a polysaccharide, the adoption of their use could still make them perceived as functional ingredients. Additionally, a pragmatic approach toward increasing the carotenoid intake could be to increase the number of bread slices consumed in a day.

The lower carotenoid concentration recorded for the participants at the end of the intervention could be attributed to factors including impaired bioaccessibility and bioavailability of the carotenoids due to the matrix type used for the carotenoid delivery in this study. This is because earlier studies (Martínez‐Tomás et al., 2012; Müller et al., 1999) that used liquid‐based food matrix for carotenoid delivery recorded significant increase in carotenoid concentration after the intervention period. In the study by Martínez‐Tomás et al. (2012), 300 mL of soup formulated from tomato, broccoli, and carrots was fed to the study participants. The concentration of carotene and lycopene present in the soup were respectively 3.9 and 4 mg. The authors observed a significant increase in serum β‐carotene concentration over the baseline amounts after 3 weeks (0.33 vs. 0.69 μmol/L) and 4 weeks (0.69 vs. 0.78 μmol/L) of the soup intake (Martínez‐Tomás et al., 2012). In another study, where tomato and carrot juice was consumed with the main diet of the participants over a 2‐week period, an 8.6‐ and 3.2‐fold increase in plasma α‐ and β‐carotene concentrations were recorded, respectively (Müller et al., 1999). The null findings observed for this present study could be due to the apparently higher concentrations of β‐carotenes in the liquid‐based foods from the two studies (juices and soup) compared to β‐carotene concentration of 237 μg/100 g present in our vegetable‐enriched bread.

Lower vegetable and fruit intake than the New Zealand daily recommendations of at least two servings of fruit and five servings of vegetables was recorded for the participants in the present study (Ministry of Health, 2022). Interestingly, the correlation matrix revealed that carotenoid reflection score and plasma carotenoid score were positively associated with the total carotenoids from the carotenoid‐rich foods. In New Zealand, only 39% of adults aged 15 years and over meet the recommended two fruits and three vegetables a day according to the Ministry of Health reports (Ministry of Health, 2011). Participants' median daily intake was low and a number of participants reported no intake of carotenoid foods. In New Zealand, cost and access are important factors that drive the low participants' consumption of vegetables and fruit (Metcalf et al., 2014), especially for low‐income families. For example, to meet the 5+ a day recommendation for a family of six will require they consume 21 kg of vegetables and fruits per week which will financially create a burden on the family. Consequent to this, stakeholders and decision‐makers in the public health space will need to implement strategic pragmatic approaches geared toward promoting increased public intake of vegetables and fruits in Australia where there is no goods and services tax on vegetables and fruits purchased.

### 4.1 Strength and limitations

The use of a novel vegetable‐enriched bread using local ingredients to increase vegetable intake is innovative. This study adds to knowledge of the feasibility of interventions aimed at increasing body carotenoid stores and validates further a non‐invasive method to measure carotenoid status, and vegetable and fruit intake. The laboratory spectrophotometry measure and the Veggie Meter™ measure use similar wavelengths to detect a number of carotenoids including lycopene. They are, however, detecting carotenoid concentration in different tissues, plasma, and fat, respectively, so there may be differences, particularly following a meal. In future studies, we would include tomato‐based foods, for example, pizza and tomato sauce in the carotenoid food questionnaire. The sample size of 10 was small, and future research should include a larger sample size and dose relative to body weight. Carotenoid uptake following bread consumption and its storage status in the body could be determined using the Veggie meter™ as it measures the composite score of all the carotenoids deposited in the fat pad of the skin (Ermakov et al., 2018).

## 5 CONCLUSION

The consumption of vegetable‐enriched bread (200 g/day) for a 2‐week period did not significantly alter participants' carotenoid status. The validation of the non‐invasive portable Veggie meter™ carotenoid reflection score against the invasive plasma carotenoid concentration suggests the Veggie meter™ has the potential to be a useful device for large population‐based studies to investigate vegetable and fruit intake. The feasibility of the application of the Veggie Meter™ and the short carotenoid foods intake questionnaire for larger population‐based studies is promising.

## CONFLICT OF INTEREST STATEMENT

There is no conflict of interest.

## Data Availability

The data that support the findings of this study are available from the corresponding author upon reasonable request.
